# Occurrence of Left Ventricular Cavity Obliteration

**DOI:** 10.7759/cureus.107281

**Published:** 2026-04-18

**Authors:** K Tamilselvan, Aashish Arumugam, Magesh Vadivelu

**Affiliations:** 1 Department of Cardiology, Meenakshi Medical College and Research Institute, Kanchipuram, IND

**Keywords:** dilated cardiomyopathy, echocardiography, hypertrophic obstructive cardiomyopathy, intracavitary gradient, left ventricular cavity obliteration, left ventricular gradient

## Abstract

Background: Left ventricular cavity obliteration (LVCO) is an echocardiographic phenomenon characterized by near-complete systolic apposition of the ventricular walls with associated intracavitary flow acceleration and gradient formation. Although often discussed in relation to hypertrophic phenotypes, its clinical profile and diagnostic spectrum among patients with left ventricular (LV) intracavitary gradients remain insufficiently described. This study evaluated the occurrence and echocardiographic predictors of LVCO in patients with Doppler-detected LV cavity gradients.

Methods: This hospital-based prospective observational cross-sectional study was conducted in the Department of Cardiology at Meenakshi Medical College Hospital and Research Institute, Kanchipuram, India, from November 2024 to December 2025. Sixty consecutive adults with a peak Doppler LV intracavitary gradient of at least 30 mmHg on transthoracic echocardiography were included. LVCO was defined using integrated two-dimensional and Doppler criteria. Associations with diagnostic category were assessed using the Pearson chi-square test, cavity size differences using the independent-samples t-test, and predictors of LVCO using binary logistic regression.

Results: The mean age of the cohort was 48.87 ± 18.88 years; 25 patients (41.7%) were male and 35 (58.3%) were female. LVCO was present in 31 of 60 patients (51.7%). Dilated cardiomyopathy was the most frequent diagnosis overall (20/60, 33.3%), followed by hypertrophic obstructive cardiomyopathy (17/60, 28.3%). The distribution of diagnostic categories did not differ significantly according to LVCO status (χ²(3) = 3.90, p = 0.272). Among 50 patients with available cavity measurements, mean LV cavity size was comparable between groups (37.39 ± 0.67 mm vs 37.18 ± 0.81 mm; t(48) = 1.00, p = 0.323). On logistic regression, hypertension showed a non-significant trend toward higher odds of LVCO (odds ratio [OR] 5.30, 95% confidence interval [CI] 0.97-29.0, p = 0.054), whereas the peak baseline LV gradient showed a statistically significant but modest inverse association with LVCO (OR 0.97 per 1 mmHg increase, 95% CI 0.94-0.99, p = 0.030). The model showed acceptable apparent discrimination and calibration within the study sample, with an area under the curve of 0.80, Hosmer-Lemeshow statistic of 6.20 (p = 0.61), and Nagelkerke R² of 0.32; however, the discriminative estimate may be optimistic given the modest sample size and lack of validation.

Conclusions: LVCO was a frequent finding in patients with echocardiographic LV intracavitary gradients but was not significantly associated with a single diagnostic category. These findings suggest that, in this selected hospital-based cohort of patients with measurable intracavitary gradients, LVCO may be better interpreted as a dynamic hemodynamic pattern rather than a diagnosis-specific marker; however, this inference should be considered preliminary and hypothesis-generating.

## Introduction

Left ventricular (LV) intracavitary gradients are clinically important yet often mechanistically heterogeneous findings on echocardiography. Although commonly discussed in relation to hypertrophic cardiomyopathy, such gradients do not uniformly indicate classical left ventricular outflow tract (LVOT) obstruction. In hypertrophic cardiomyopathy, obstruction is a major determinant of symptoms and prognosis, with resting or provoked gradients occurring in a substantial proportion of patients and higher resting gradients carrying adverse clinical significance [1-4]. However, the mere presence of an LV gradient does not define its anatomical level or physiological basis.

Left ventricular cavity obliteration (LVCO) represents a distinct phenomenon characterized by near-complete systolic apposition of the ventricular walls, usually at the mid-ventricular or apical level, producing intracavitary flow acceleration and a late-peaking Doppler signal. This pattern may resemble obstructive hypertrophic cardiomyopathy, yet prior invasive, angiographic, and echocardiographic studies have shown that cavity obliteration can occur independently of true subaortic obstruction and should not be interpreted as an equivalent entity [5-7]. Its occurrence has been linked to small hyperdynamic ventricles, increased wall thickness, altered loading conditions, and stress-related physiological states [8,9].

The significance of LVCO becomes particularly relevant in hypertrophic phenotypes, where mid-cavity or apical obliteration has been associated with ventricular arrhythmia, apical ischemia, aneurysm formation, embolic events, and adverse cardiovascular outcomes [10-12]. At the same time, multimodality studies have shown that several dynamic gradients may coexist in the same patient, making precise echocardiographic interpretation essential for accurate diagnosis and management [13-15].

Despite growing recognition of this entity, prospective data describing the clinical profile, diagnostic spectrum, and echocardiographic correlates of LVCO among patients with LV cavity gradients remain limited. This gap is relevant because such patients may represent a heterogeneous population rather than a single disease phenotype. Therefore, the present study was undertaken to evaluate the echocardiographic predictors of LVCO among patients with echocardiographic LV cavity gradients.

## Materials and methods

This hospital-based prospective observational cross-sectional study was conducted in the Department of Cardiology at Meenakshi Medical College Hospital and Research Institute Enathur Kanchipuram over a 14-month period from Nov 2024 to Dec 2025. Institutional Ethics Committee approval was obtained before commencement of the study (MMCHRI/IEC/Faculty/22 Oct 2024 dated 14 Oct 2024). The study was designed to evaluate the clinical profile, diagnostic spectrum, and echocardiographic predictors of LVCO among patients demonstrating echocardiographic LV cavity gradients.

Study population

Consecutive adult patients aged 18 years and above who demonstrated a peak Doppler LV intracavitary gradient of ≥30 mmHg on transthoracic echocardiography during the study period were screened for eligibility. This threshold was used to ensure inclusion of patients with clearly measurable intracavitary gradients, but it also selected a specific subgroup and may limit applicability to patients with milder, intermittent, or provoked gradients. Patients were excluded if they were younger than 18 years, if they declined participation, if the acoustic window was inadequate for reliable assessment of cavity obliteration, or if the intracavitary gradient could not be measured accurately. A total of 60 eligible patients were included in the final analysis.

Clinical assessment

All participants underwent prospective clinical evaluation using a structured case record form. Demographic variables including age and sex were recorded. Clinical details included presenting symptoms and major comorbidities with particular attention to hypertension and diabetes mellitus. Presenting complaints such as palpitations and dyspnea were documented at the time of assessment. Final diagnostic categorization was established after integrated clinical and echocardiographic evaluation using predefined criteria. Hypertrophic obstructive cardiomyopathy (HOCM) was diagnosed in patients with unexplained LV hypertrophy and dynamic obstruction consistent with hypertrophic cardiomyopathy. Dilated cardiomyopathy was diagnosed in patients with LV dilatation accompanied by reduced systolic function. A normal study was assigned when no overt clinically meaningful structural or functional cardiac abnormality was identified on routine echocardiographic and clinical assessment, despite the presence of an intracavitary gradient; this category may therefore have included physiologic dynamic flow phenomena or subclinical findings not otherwise classifiable within the predefined diagnostic groups. The other diagnoses category included patients who did not fulfill the predefined criteria for HOCM, dilated cardiomyopathy or normal study.

Echocardiographic evaluation

All patients underwent transthoracic echocardiography using a Philips EPIQ CVx ultrasound system (Philips Ultrasound LLC, Bothell, WA, USA). Standard two-dimensional imaging, color Doppler, pulsed-wave Doppler, and continuous-wave Doppler were performed from standard parasternal and apical views in accordance with American Society of Echocardiography recommendations for comprehensive transthoracic echocardiography and chamber assessment. Peak LV intracavitary gradient was identified and measured by continuous-wave Doppler after careful alignment with the site of maximal intracavitary flow acceleration. Wherever feasible, two-dimensional and color Doppler imaging were used to localize the level of flow acceleration and to distinguish intracavitary or midventricular acceleration from classical LVOT obstruction. LVCO was defined using integrated two-dimensional and Doppler criteria as near-complete systolic apposition of opposing LV walls at the midventricular or apical level unrelated to systolic anterior motion, with minimal residual distal cavity on two-dimensional imaging and a corresponding intracavitary Doppler signal or gradient. For analysis, LVCO was recorded as a binary variable as present or absent. Peak baseline LV gradient was documented as a continuous variable. LV cavity size in millimeters was recorded from the baseline echocardiographic study, wherever available. Since cavity size measurements were not available in all patients, analyses involving this variable were restricted to cases with complete data. Echocardiographic studies were interpreted by one experienced cardiologist with expertise in transthoracic echocardiography. Blinding was not performed. Because image interpretation was conducted by a single observer, formal interobserver reproducibility assessment could not be undertaken, and potential observer-dependent classification bias cannot be excluded.

Study variables and outcome measures

The primary outcome variable was the presence of LVCO. Secondary analyses included estimation of the overall frequency of LVCO within the cohort, description of diagnostic categories, assessment of the association between LVCO status and diagnostic category, comparison of mean LV cavity size between patients with and without LVCO, and evaluation of selected clinical and echocardiographic predictors of LVCO.

Statistical analysis

Data were entered in Microsoft Excel and analyzed using IBM SPSS Statistics for Windows, Version 25 (Released 2017; IBM Corp., Armonk, New York, United States). Continuous variables were expressed as mean ± standard deviation, and categorical variables were summarized as n (%). Normality of continuous variables was assessed using the Shapiro-Wilk test together with visual inspection of histograms and/or Q-Q plots before application of parametric tests. Baseline demographic and clinical characteristics, frequency of LVCO, and diagnostic distribution were described using descriptive statistics. The association between LVCO status and diagnostic category was assessed using the Pearson chi-square test. Mean LV cavity size was compared between patients with and without LVCO using the independent-samples t-test. Because complete cavity-size data were available in only 50 patients, this comparison was performed using a complete-case approach. The pattern of missingness was not formally modeled; therefore, whether the missing data were random could not be established.

Binary logistic regression analysis was performed to identify predictors of LVCO. Given the modest sample size and the limited number of LVCO events, a parsimonious exploratory model was constructed to reduce the risk of overfitting; however, this approach does not eliminate the possibility of residual confounding or coefficient instability. Hypertension and peak baseline LV gradient were selected a priori on clinical grounds. Additional potential confounders, including age, sex, diabetes mellitus, and diagnostic category, were considered but were not retained because the limited events-per-variable ratio and sparse subgroup frequencies constrained model complexity. Accordingly, the regression analysis should be interpreted as exploratory rather than definitive. Logistic regression results are reported as regression coefficient (B), standard error (SE), Wald chi-square statistic, odds ratio (OR), 95% confidence interval (CI), and p-value. Model calibration was assessed using the Hosmer-Lemeshow goodness-of-fit test, and model explanatory performance was summarized using Nagelkerke R², while model discrimination was assessed using receiver operating characteristic curve analysis and the area under the curve (AUC). A p-value of less than 0.05 was considered statistically significant.

## Results

A total of 60 patients with echocardiographic LV cavity gradients were included in this study. The mean age of the cohort was 48.87 ± 18.88 years, with an age range of 18-80 years. Of the 60 participants, 25 (41.7%) were male and 35 (58.3%) were female. Hypertension was the most common comorbidity and was present in 44 (73.3%) patients, whereas diabetes mellitus was noted in 15 (25.0%) patients. Palpitations and dyspnea were the most frequent presenting symptoms, reported in 13 (21.7%) and 12 (20.0%) patients, respectively (Table 1).

**Table 1:** Baseline demographic and clinical characteristics of the study population (n = 60) Data are presented as mean ± standard deviation or n (%).

Variable	Value
Age (years), mean ± SD	48.87 ± 18.88
Age range (years)	18-80
Male, n (%)	25 (41.7)
Female, n (%)	35 (58.3)
Hypertension, n (%)	44 (73.3)
Diabetes mellitus, n (%)	15 (25.0)
Palpitations, n (%)	13 (21.7)
Dyspnea, n (%)	12 (20.0)

LVCO was present in 31 (51.7%) patients, while 29 (48.3%) did not demonstrate LVCO. Thus, LVCO was identified in slightly more than half of the study population (Table 2).

**Table 2:** Occurrence of left ventricular cavity obliteration among study participants (n = 60) Data are presented as n (%). LVCO: left ventricular cavity obliteration.

LVCO status	n (%)
Present	31 (51.7)
Absent	29 (48.3)

With respect to diagnostic distribution, dilated cardiomyopathy was the most frequent diagnosis overall and was observed in 20 (33.3%) patients, followed by HOCM in 17 (28.3%). Thirteen patients (21.7%) had normal findings, while 10 (16.7%) were classified under other diagnoses. In this context, the normal category denotes the absence of overt structural or functional abnormality on routine clinical and echocardiographic assessment rather than the absence of a measurable intracavitary gradient (Table 3).

**Table 3:** Differential diagnosis among study participants (n = 60) Data are presented as n (%).

Diagnosis	n (%)
Dilated cardiomyopathy	20 (33.3)
Hypertrophic obstructive cardiomyopathy	17 (28.3)
Normal	13 (21.7)
Other	10 (16.7)

When LVCO status was compared across diagnostic categories, the distribution differed numerically but did not reach statistical significance on Pearson chi-square testing (χ² (3) = 3.90, p = 0.272). HOCM was relatively more frequent in the LVCO-present group, whereas dilated cardiomyopathy was relatively more frequent in the LVCO-absent group. However, the overall diagnostic distribution was not significantly different between the two groups (Table 4).

**Table 4:** Association between left ventricular cavity obliteration and differential diagnosis Data are presented as frequency counts. Statistical test used: Pearson chi-square test, χ²(3) = 3.90, p = 0.272. LVCO: left ventricular cavity obliteration.

Diagnosis	LVCO absent, n = 29	LVCO present, n = 31
Dilated cardiomyopathy	11	9
Hypertrophic obstructive cardiomyopathy	6	11
Normal	5	8
Other	7	3

Among the 50 patients with available LV cavity size measurements, the mean LV cavity size was 37.39 ± 0.67 mm in the LVCO-present group and 37.18 ± 0.81 mm in the LVCO-absent group. This difference was not statistically significant on an independent-samples t-test (t (48) = 1.00, p = 0.323), indicating that the mean LV cavity size was comparable between the two groups within the complete-case subset; however, this finding should be interpreted cautiously because non-random missingness cannot be excluded (Table 5).

**Table 5:** Comparison of mean left ventricular cavity size between patients with and without left ventricular cavity obliteration Data are presented as mean ± standard deviation. Statistical test used: independent-samples t-test, t(48) = 1.00, p = 0.323. LVCO: left ventricular cavity obliteration.

Group	n	Mean ± SD (mm)
LVCO present	25	37.39 ± 0.67
LVCO absent	25	37.18 ± 0.81

In binary logistic regression analysis, hypertension showed a non-significant trend toward higher odds of LVCO (B = 1.668, SE = 0.867, Wald χ² = 3.70, OR 5.30, 95% CI 0.97-29.0, p = 0.054). The peak baseline LV gradient showed a statistically significant but modest inverse association with LVCO status (B = -0.030, SE = 0.013, Wald χ² = 5.31, OR 0.97 per 1 mmHg increase, 95% CI 0.94-0.99, p = 0.030). Additional candidate confounders, including age, sex, diabetes mellitus, and diagnostic category, were considered during model construction but were not retained because of the modest sample size, limited number of outcome events, and sparse subgroup frequencies. The resulting regression estimates should therefore be interpreted as exploratory and potentially vulnerable to residual confounding (Table 6).

**Table 6:** Predictors of left ventricular cavity obliteration Statistical test used: binary logistic regression analysis. OR for peak baseline left ventricular gradient is expressed per 1 mmHg increase. Statistical significance was set at p < 0.05.

Predictor	B	SE	Wald χ²	OR (Exp B)	95% CI	p-value
Hypertension	1.668	0.867	3.70	5.30	0.97-29.0	0.054
Peak baseline LV gradient (per 1 mmHg increase)	-0.030	0.013	5.31	0.97	0.94-0.99	0.030

The logistic regression model showed an overall accuracy of 76.7%, with a sensitivity of 77.4% and specificity of 75.9%. The area under the receiver operating characteristic curve was 0.80, suggesting acceptable apparent discriminative ability within the study sample; however, this estimate may be optimistic given the modest sample size and lack of validation. Model calibration was also acceptable, with a Hosmer-Lemeshow goodness-of-fit statistic of 6.20 (p = 0.61) and a Nagelkerke R² of 0.32 (Table 7).

**Table 7:** Performance characteristics of the logistic regression model for predicting left ventricular cavity obliteration Statistical methods used: binary logistic regression classification analysis and receiver operating characteristic curve analysis. AUC: area under the curve; ROC: receiver operating characteristic.

Parameter	Value
Accuracy	76.7%
Sensitivity	77.4%
Specificity	75.9%
AUC (ROC curve)	0.80
Hosmer–Lemeshow χ², p	6.20, 0.61
Nagelkerke R²	0.32

## Discussion

The present study advances a practical point that may be easy to miss in day-to-day echocardiography: LVCO is common among patients with measurable intracavitary gradients, but it does not map neatly onto a single diagnostic label. In this cohort, LVCO was identified in 51.7% of patients, yet its distribution across dilated cardiomyopathy, HOCM, normal studies, and other diagnoses was not statistically different. Mean LV cavity size also did not differ significantly between LVCO-positive and LVCO-negative groups. In multivariable analysis, hypertension showed only a non-significant trend toward higher odds of LVCO, whereas peak baseline LV gradient demonstrated a statistically significant but quantitatively modest inverse association with LVCO, the clinical significance of which remains uncertain, and the final model showed acceptable apparent discrimination and calibration within the derivation cohort, although these performance estimates may be optimistic. Taken together, these findings suggest that, within this selected hospital-based cohort, LVCO may be interpreted as a dynamic hemodynamic pattern rather than a diagnosis-specific marker; however, this interpretation should be considered exploratory and hypothesis-generating rather than definitive.

This interpretation is broadly aligned with earlier mechanistic literature. Raizner et al. (1977) [5] described cavity obliteration across a wider hypertrophic and pressure-overload spectrum rather than as an exclusive correlate of classical obstructive hypertrophic cardiomyopathy. Sobrino et al. (1980) [6] similarly showed that cavity obliteration may have a hemodynamic behavior distinct from dynamic subaortic obstruction. More recently, Pollick et al. (2020) [7] demonstrated that LVCO generates an intracavitary gradient whose magnitude relates to the extent and duration of cavity closure and that this physiology should be differentiated from HOCM even when the Doppler envelope appears late systolic and dagger-shaped. The current study is concordant with that concept: although HOCM was numerically more frequent in the LVCO-present group, the absence of a significant association with final diagnosis argues against equating LVCO with one structural disease category.

At the same time, some findings diverge from parts of the published literature and deserve a measured interpretation. Khanal et al. (1998) [9] reported that stress-induced cavity obliteration was associated with smaller ventricular dimensions, greater wall thickness, and hyperdynamic function, particularly in women. Leung and Levine (1994) [8] likewise linked end-systolic cavity obliteration to reduced ventricular filling in the intraoperative setting. By contrast, the present study did not detect a significant difference in mean cavity size between groups. This discrepancy is plausible for several reasons. First, prior studies often examined provoked physiology during dobutamine stress or intraoperative preload shifts, where chamber underfilling and hypercontractility are accentuated. The present study evaluated a resting clinical cohort with pre-existing intracavitary gradients. Second, the cohort was diagnostically heterogeneous, which likely diluted phenotype-specific geometric patterns. Third, cavity size was represented by a single linear measure rather than a comprehensive volumetric, regional, or three-dimensional assessment, which may have reduced sensitivity for detecting subtle geometric distinctions relevant to LVCO.

The inverse association between peak baseline gradient and LVCO may also appear counterintuitive if one assumes that more obliteration should always yield a larger gradient. However, that assumption is overly simplistic. As emphasized by Elsayes et al. (2018) [14] and de Gregorio et al. (2006) [13], multiple dynamic gradients may coexist within the same ventricle, and their timing and anatomical level matter greatly. In a mixed cohort such as this one, a higher Doppler gradient may in some patients reflect outflow tract or mixed intraventricular physiology rather than more complete cavity apposition alone. Accordingly, this finding should be interpreted cautiously. Given the small effect size, the heterogeneous substrate of the cohort, and the cross-sectional design, the observed inverse relationship is better regarded as an exploratory statistical signal than as evidence of a definitive mechanistic pathway.

The clinical implications are important. LVCO should not be dismissed as a benign echocardiographic curiosity, nor should it be reflexively labeled as obstructive HCM. Studies in apical and mid-cavity hypertrophic phenotypes have linked sustained obliteration to ischemia, paradoxical jet flow, apical aneurysm formation, embolic phenomena, arrhythmia, and adverse outcomes, as shown by Matsubara et al. (2003) [10], Kim et al. (2016) [11], and Nakamura et al. (1992) [16]. Yet those risks arise in specific phenotypic contexts. The practical lesson from the present data is that once a ventricular gradient is detected, the sonographer and clinician must define its site, timing, morphology, and structural substrate before drawing diagnostic or therapeutic conclusions. In that sense, the present findings favor a hemodynamic and anatomically localized interpretation of ventricular gradients rather than a purely label-driven one.

This study has several limitations. It was a single-center investigation with a modest sample size, which constrained subgroup comparisons and limited the robustness of multivariable modeling. Because the study was cross-sectional and hospital-based, the findings do not permit causal inference and may not be generalizable to broader echocardiographic populations, particularly community-based or unselected referral cohorts. The population was diagnostically heterogeneous, and this likely diluted stronger phenotype-specific associations. In addition, the requirement for a baseline intracavitary gradient of at least 30 mmHg enriched the cohort for a selected physiological subset and may limit extrapolation to general echocardiographic populations. LV cavity size was available in only 50 of the 60 patients, and analyses involving this variable were therefore restricted to complete cases. Because the mechanism of missingness was not formally assessed, selection bias related to missing data cannot be excluded. In addition, cavity size was assessed using a linear measurement rather than a fuller geometric or volumetric analysis. Echocardiographic interpretation was performed by a single experienced cardiologist without blinding, and formal interobserver or intraobserver reproducibility assessment was not performed. This represents an important methodological limitation because observer-dependent classification bias and uncertainty regarding reproducibility cannot be excluded. In addition, the study did not include provocation testing, contrast echocardiography, cardiac magnetic resonance imaging, or longitudinal follow-up, which limits mechanistic clarification and prevents prognostic inference.

## Conclusions

LVCO was a frequent echocardiographic finding in patients with LV intracavitary gradients, but it was not significantly associated with a single diagnostic category. The results suggest that, in this selected cohort of patients with measurable intracavitary gradients, LVCO may be better understood as a dynamic hemodynamic pattern than as a diagnosis-specific marker; however, this interpretation should be viewed as preliminary and hypothesis-generating. Careful anatomical localization of the gradient and integrated interpretation of two-dimensional and Doppler findings remains essential to avoid misclassification, particularly as classical LVOT obstruction. Larger prospective studies with standardized imaging protocols, blinded assessment, reproducibility analysis, multimodality correlation, and outcome follow-up are needed to define the full diagnostic and clinical significance of LVCO more precisely.

## References

[1] Varma PK, Neema PK (2014). Hypertrophic cardiomyopathy: part 1 - introduction, pathology and pathophysiology. Ann Card Anaesth.

[2] Elliott PM, Anastasakis A, Borger MA (2014). 2014 ESC Guidelines on diagnosis and management of hypertrophic cardiomyopathy: the Task Force for the Diagnosis and Management of Hypertrophic Cardiomyopathy of the European Society of Cardiology (ESC). Eur Heart J.

[3] Maron MS, Olivotto I, Betocchi S (2003). Effect of left ventricular outflow tract obstruction on clinical outcome in hypertrophic cardiomyopathy. N Engl J Med.

[4] Todde G, Canciello G, Borrelli F, Perillo E, Esposito G, Lombardi R, Losi M (2023). Diagnosis and treatment of obstructive hypertrophic cardiomyopathy. Cardiogenetics.

[5] Raizner AE, Chahine RA, Ishimori T, Awdeh M (1977). Clinical correlates of left ventricular cavity obliteration. Am J Cardiol.

[6] Sobrino JA, Lanchas CH, Del Rio A, Maté I, Carrillo A, Imizcoz MA, Sobrino N (1980). Left ventricular cavity obliteration: hemodynamic behavior of the postextrasystolic beat. Am Heart J.

[7] Pollick C, Shmueli H, Maalouf N, Zadikany RH (2020). Left ventricular cavity obliteration: mechanism of the intracavitary gradient and differentiation from hypertrophic obstructive cardiomyopathy. Echocardiography.

[8] Leung JM, Levine EH (1994). Left ventricular end-systolic cavity obliteration as an estimate of intraoperative hypovolemia. Anesthesiology.

[9] Khanal S, Daggubati R, Gaalla A, Shah PM, Pai RG (1998). Left ventricular cavity obliteration during dobutamine stress echocardiography is associated with female sex and left ventricular size and function. J Am Soc Echocardiogr.

[10] Matsubara K, Nakamura T, Kuribayashi T, Azuma A, Nakagawa M (2003). Sustained cavity obliteration and apical aneurysm formation in apical hypertrophic cardiomyopathy. J Am Coll Cardiol.

[11] Kim H, Park JH, Won KB (2016). Significance of apical cavity obliteration in apical hypertrophic cardiomyopathy. Heart.

[12] Perillo EF, Canciello G, Borrelli F (2023). Diagnosis and clinical implication of left ventricular aneurysm in hypertrophic cardiomyopathy. Diagnostics (Basel).

[13] de Gregorio C, Recupero A, Grimaldi P, Coglitore S (2006). Can transthoracic live 3-dimensional echocardiography improve the recognition of midventricular obliteration in hypertrophic obstructive cardiomyopathy?. J Am Soc Echocardiogr.

[14] Elsayes AH, Joshi B, Wessler B, Rowin EJ, Maron MS, Cobey FC (2018). A case of multiple ventricular gradients. J Cardiothorac Vasc Anesth.

[15] Webb JJ (2024). Mid-ventricular obliteration diagnosed by bedside-focused echocardiography in the emergency department in a patient with syncope and associated hypertrophic cardiomyopathy: a case report. Cureus.

[16] Nakamura T, Matsubara K, Furukawa K, Azuma A, Sugihara H, Katsume H, Nakagawa M (1992). Diastolic paradoxic jet flow in patients with hypertrophic cardiomyopathy: evidence of concealed apical asynergy with cavity obliteration. J Am Coll Cardiol.

